# Crop yield response to climate change varies with crop spatial distribution pattern

**DOI:** 10.1038/s41598-017-01599-2

**Published:** 2017-05-03

**Authors:** Guoyong Leng, Maoyi Huang

**Affiliations:** 1 Joint Global Change Research Institute, Pacific Northwest National Laboratory, College Park MD, Riverdale, 20740 USA; 2 Earth System Analysis and Modeling Group, Atmospheric Sciences & Global Change Division, Pacific Northwest National Laboratory, Riverdale, USA

## Abstract

The linkage between crop yield and climate variability has been confirmed in numerous studies using statistical approaches. A crucial assumption in these studies is that crop spatial distribution pattern is constant over time. Here, we explore how changes in county-level corn spatial distribution pattern modulate the response of its yields to climate change at the state level over the Contiguous United States. Our results show that corn yield response to climate change varies with crop spatial distribution pattern, with distinct impacts on the magnitude and even the direction at the state level. Corn yield is predicted to decrease by 20~40% by 2050 s when considering crop spatial distribution pattern changes, which is 6~12% less than the estimates with fixed cropping pattern. The beneficial effects are mainly achieved by reducing the negative impacts of daily maximum temperature and strengthening the positive impacts of precipitation. Our results indicate that previous empirical studies could be biased in assessing climate change impacts by ignoring the changes in crop spatial distribution pattern. This has great implications for understanding the increasing debates on whether climate change will be a net gain or loss for regional agriculture.

## Introduction

Understanding the relationship between climate and crop yield helps in enhancing resilience of agricultural production systems to climate change. Towards this, numerous studies have been conducted investigating how crop yield respond to climate conditions, with emphasis on the decadal and interannual climate variability^1–3^, climate extremes^4–7^, vapor pressure deficits^8^, atmospheric CO_2_ concentration^9^, as well as climate covariability^10^. On the negative side, extreme weather events such as drought have exhibited an increasing trend^11–13^ and resulted in declined crop production^4, 6, 14^. To provide key information for early warning and mitigation strategies, drought monitoring and prediction system has been designed to improve crop yield and resilience against droughts^15–17^. At the same time, adaptation measures such as shifting planting dates, switching to an existing crop variety, development of new crop varieties as well as changes in crop growing pattern may modulate negative impacts and shape the severity of climate impacts on crop yield and production^18–27^. Indeed, farmers tend to grow crop in lands with optimal water and nutrient storage conditions, resulting in subsequent changes in crop spatial distribution pattern. Hence, a key question arise to how historical changes in crop spatial distribution pattern have regulated crop yield response to climate change?

Empirical models that rely on past observations of climate and crop yields are recognized as efficient tools and have been extensively used in previous studies^28^. Typically, to derive climate indices associated with crops grown in a region, gridded climate values are averaged over crop growing areas with weights based on gridded crop area map^29, 30^. Nearly all of previous empirical studies assumed that the relative distribution of crops in space has remained constant over the study period^1, 31–39^ since fine-scale datasets of crop area and the changes do not yet exist. However, it is likely that for some crop–region combinations this may not be valid due to shift in spatial distribution pattern made possible by advances in crop technology, competing land use and etc. Therefore, investigating the effects of crop spatial distribution pattern changes can not only help explore the implications of its adaption role in modulating historical climate impacts on yields, but also understanding the uncertainty in projecting future crop yields.

Here, we provide a data-driven analysis to investigate the role of changes in crop spatial distribution pattern between counties in regulating corn yield response to climate change at the state level over Contiguous United States (CONUS). By comparing the estimates of climate impacts on crop yield with and without accounting for crop spatial distribution pattern changes, uncertainties by assuming constant crop spatial distribution pattern as in previous empirical studies can be explored. This also helps understanding the increasing debates on whether warming will be a net gain or loss for agriculture regions like that of CNOUS by strengthening our understanding on this least understood uncertainty source. Specifically, we will address the following two scientific questions: (1) how changes in crop spatial distribution pattern have regulated the response of crop yield to historical climate change over CONUS? and (2) How much uncertainty would be expected when projecting future climate change impacts if assuming fixed crop spatial distribution pattern? Although we focus our analyses on corn in the CONUS, which account for ca. 41% of the world’s total production, the analysis framework can be extended to other crops of interest. Our results are conducted at the state-level since county-level corn area and yield information is available over CONUS.

## Results

### Changes in corn area and spatial distribution pattern

**Figure 1:**
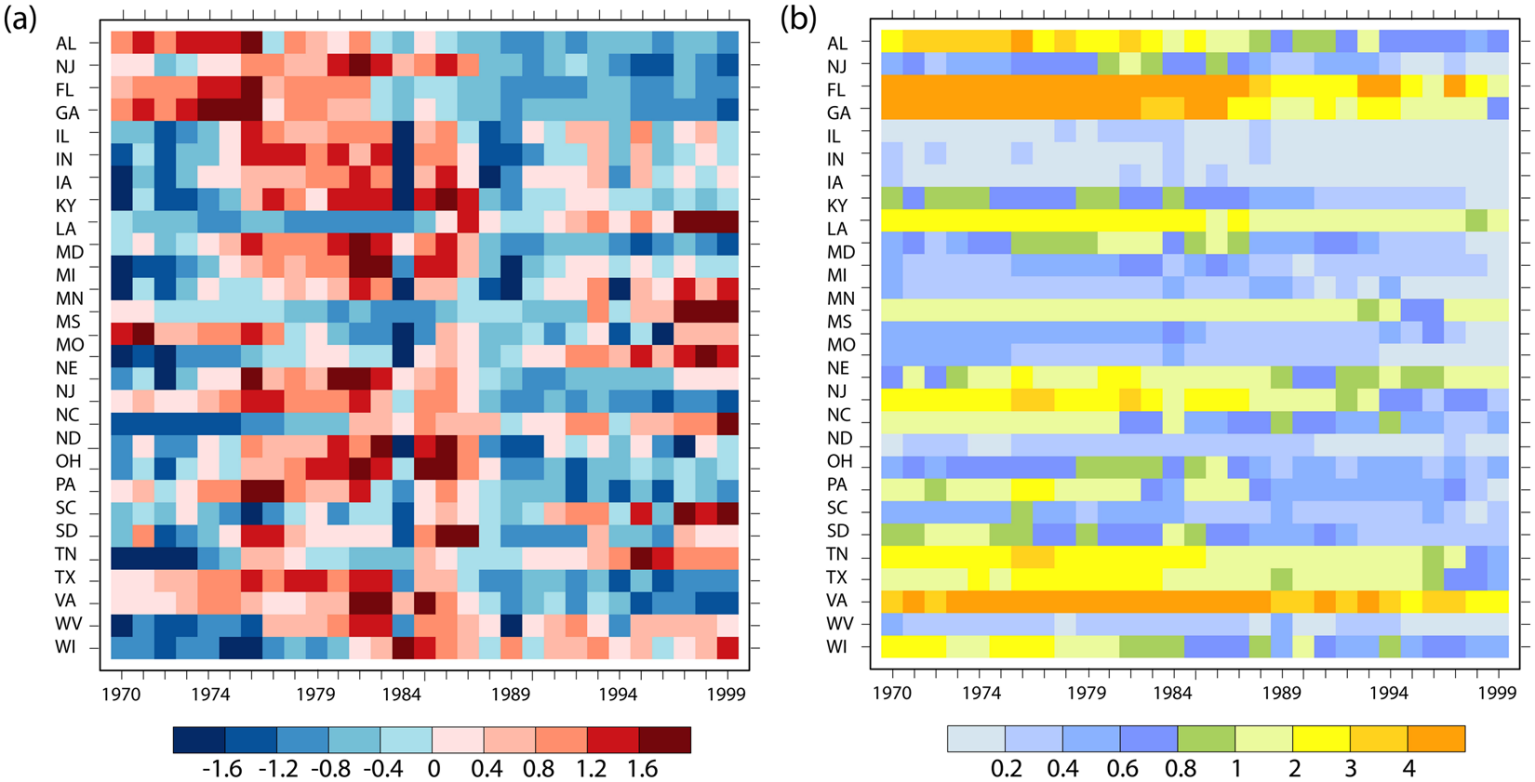
(**a**) changes in annual corn area anomaly (divided by the standardard deviation) relative to the long-term mean and (**b**) changes in *SI*_*s*_, with higher *SI*_*s*_ indicating larger instability of crop growing pattern and vice versa. Note: only states where the constructed empirical model is significant at the 95% confidence level are included for analysis (see Table 1). Figure was created by NCAR Command Language^57^.

Figure 1 shows the evolution of corn growing area anomalies and the stability index (*SI*_*s*_) indicating spatial distribution pattern changes in those states where statistically significant relations between climate and corn yield are constructed. Corn area has changed significantly with anomaly larger than one standard deviation for most states (Fig. 1a). Specifically, annual corn areas exhibited an increasing trend before the year 1985 followed by decrease and then peaked in some states. Most of the increase in corn area concentrated during the period 1975–1990 with anomaly larger than one standard deviation, especially those high production states. Relative to the year 2000, the corn growing pattern between counties has exhibited distinct variations at the state level, with considerable changes observed in all investigated states (Fig. 1b). Such changes in corn growing pattern are more pronounced (higher *SI*_*s*_) in the southeast states and relatively small (lower *SI*_*s*_) in highly productive regions such as Illinois and Iowa.

### The role of crop spatial distribution pattern changes in modulating climate impacts on corn yield

How has change in crop spatial distribution pattern regulated corn yield response to historical climate change? Before our quantitative investigations, we first examine the performance of statistical models linking growing season precipitation (Prec), daily minimum temperature (Tmin) and daily maximum temperature (Tmax) with corn yield. Table 1 presents the portion of corn yield variance explained by the models in the investigated states. It is found that our models provided fairly accurate descriptions of historical corn yield variations, of which 40~70% can be explained by the considered climate factors at the state level. Averaged over the states, more than 60% of corn yield variance can be explained by the three climate variables. Note this does not preclude the role of other factors, but simply says that the majority of corn yield variation is driven by changes in the considered climate variables. Importantly, the portion of variance explained by the model fed with information of crop growing pattern changes is in general higher than that with fixed pattern except for MS, LA and OK. The low ability of statistical model to explain crop yield variance in MS, LA, and OK could be attributed to the fact that we only consider temperature and precipitation and their growing averages, without explicitly accounting for other factors such as extreme weathers and agricultural managements, which could largely influence crop yields (See Discussions section for details).

**Table 1:** Portion of corn yield variability explained by growing season climate variability during 1970–1999 by the empirical models with (R^2^_T_) and without (R^2^_F_) considering the changes in crop growing pattern.

State name	R^2^_T_	R^2^_F_
AL (Alabama)	61.55	62.89
DE (Delaware)	65.19	61.88
FL (Florida)	51.05	50.86
GA (Georgia)	74.49	72.31
IL (Illinois)	73.07	70.3
IN (Indiana)	69.95	66.49
IA (Iowa)	74.03	70.59
KY (Kentucky)	68.26	63.83
LA (Louisiana)	40.75	47.81
MD (Maryland)	71.39	68.41
MI (Michigan)	55.92	52.7
MN (Minnesota)	69.04	66.42
MS (Mississippi)	50.12	52.75
MO (Missouri)	63.66	58.16
NE (Nebraska)	52.68	48.71
NJ (New Jersey)	52.18	50.21
NC (North Carolina)	66.93	62.21
ND (North Dakota)	59.9	60.01
OH (Ohio)	64.2	61.74
OK (Oklahoma)	51.14	51.39
PA (Pennsylvania)	71.34	68.72
SC (South Carolina)	68.97	65.2
SD (South Dakota)	67.71	66.25
TN (Tennessee)	67.83	63.77
VA (Virginia)	79.53	72.74
WV (West Virginia)	61.37	56.6
WI (Wisconsin)	64.21	61.63
CONUS	67.10	63.01

Note: only states where the climate-corn yield relations are statistically significant at the 95% confidence level are included for investigation. The value for CONUS is the average over investigated states with weights based on the corn area.

**Figure 2:**
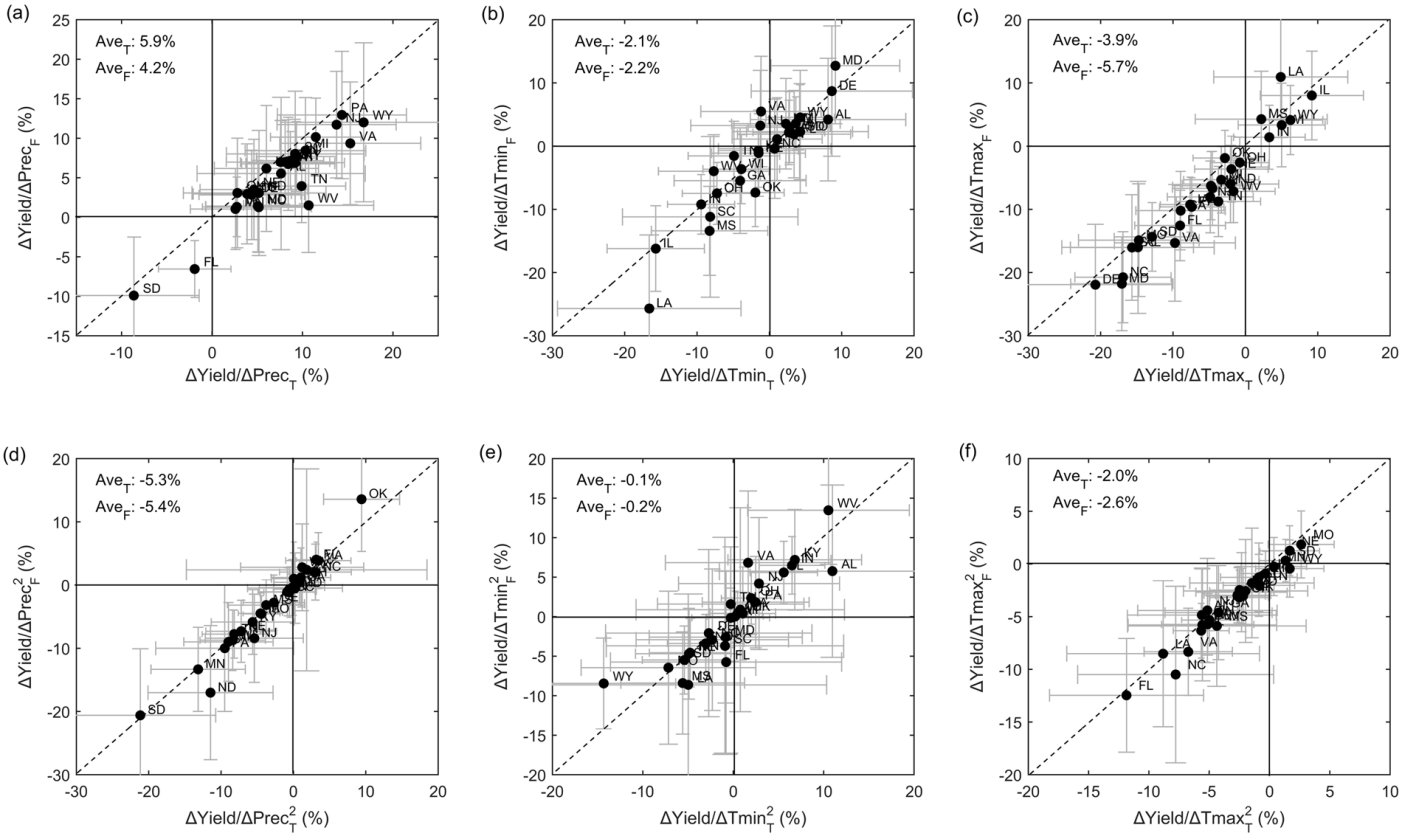
Sensitivity of corn yields to one unit increase of precipitation (Prec), daily minimum temperature (Tmin), and daily minimum temperature (Tmax) estimated by two crop models. The lower case T and F in the x and y labels indicate the estimates based on model with and without considering the changes in crop growing pattern, respectively. The first row is for the corn yield response to linear effects of climate change while the second row is for the non-linear climate effects. The sensitivity is defined as the percentage changes (%) of corn yield normalized by the long-term mean to each 1 mm/day increase of Prec, 1 °C increase of Tmin and Tmax, respectively. The number indicate the average sensitivity averaged over investigated states with weights based on the long-term mean growing area. The error bar denote the standard deviation among the 1000 samples constructed by boot-strap technique to show the crop model uncertainty ranges. Figure was created using software MATLAB 2015a (http://www.mathworks.com/).

Figure 2 shows the sensitivity of corn yields to one unit increase of each climate variable as estimated by the two statistical models. The error bar indicate the sampling uncertainty based on 1000 samples generated with bootstrap resampling technique. The regression model coefficients revealed a consistently positive response of corn yield to wet conditions in states except SD and FL (Fig. 2a). However, the positive effects of precipitation would be reversed after exceeding certain thresholds, demonstrating the non-linear climatic effects as accounted for in our model (Fig. 2d). Negative corn yield response to warmer growing season Tmin and Tmax is found for most states, with largest yield reduction up to 20% by 1° increase of temperature (e.g. in LA). More rapid crop development and greater water stress are among the most likely mechanisms behind the reduction of corn yields with climate warming. Nonetheless, a clear positive warming effect is also observed in some states possibly due to the fact that temperatures may not currently be above the optimum level for maximum photosynthesis rates in these regions, so that warming could enhance crop growth and yield^2^. Notably, corn yield exhibits distinct and even opposite sensitivity to nighttime and daytime temperatures. For example, in IL, one degree increase of Tmin leads to 16% decrease of corn yield while Tmax could enhance the corn yield by 10%. Combined, it result in negative impacts of daily mean temperature given that Tmin has risen faster than Tmax. This findings is broadly consistent with^34^ who reported negative relations between growing season daily mean temperature and corn yields in this region.

Importantly, difference between the two models are observed due to the effects of crop spatial distribution pattern changes. Our results show that corn yield response to climate change varies with crop spatial distribution pattern, with distinct impacts on the magnitude and even the direction at the state level (Fig. 2). Specifically, we found that, if not considering the changes of corn spatial distribution pattern, the positive effects of precipitation would be underestimated while the negative effects by Tmax would be enhanced. However, there are exceptions in some states, where the negative effects are enhanced with positive effects reduced after considering corn growing pattern changes. This may be due to the fact corn spatial distribution pattern is mainly determined by factors other than climate which are neglected in this study. The results demonstrate that climate change can influence crop yield not only by directly affecting the physiological response of crops to climate but also indirectly by changing the optimal land conditions and the subsequent shift of crop growing pattern, although the feasibility of these shifts would depend on a range of other factors, including topography, soils, agricultural management and competing land uses. Overall, one unit increase of precipitation lead to 5.9% increase of corn yield for the area-weighted country-mean, while the estimate is 4.2% by the model without considering crop growing pattern changes. For Tmax, the negative effects would be reduced from −5.7% to −3.9% per unit warming when the role of growing pattern is accounted for while minor effects are found in regulating the Tmin impacts. Notably, the beneficial effects of crop growing pattern changes are mainly achieved via modulating the linear climatic effects with negligible impacts on non-linear climatic effects (Fig. 2d–f).

Based on the crop model, recent climate trends have exerted an overall adverse impacts, resulting in a reduction of corn yield by 2.5% without adaptations, which is consistent with the estimates by Lobell *et al*.^1^ Although historical observations have revealed a substantial change in corn spatial distribution pattern for the study period (Fig. 1), this important factor has received limited attention to date. We show that with consideration of changing corn spatial distribution pattern, the negative climate impacts would reduce to 1.8%. Although the sampling uncertainty revealed by bootstrap resampling technique is large at the state level, our results show a clear difference in the estimated climate change impacts on corn yields at the state level by changes in crop spatial distribution pattern between counties. The reduced negative climate impacts also imply that the indirect role of climate change on corn yield through influencing crop growing pattern should be considered, although factors affecting crop growing pattern is not limited to climate conditions.

### Implications for projecting future corn yields under climate change scenarios

**Figure 3:**
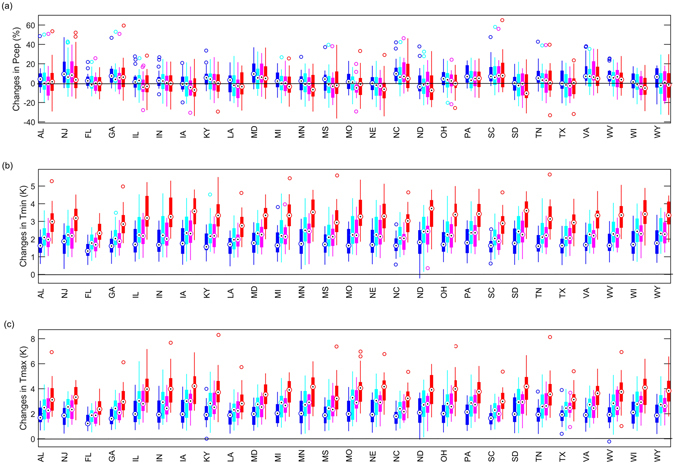
Projected changes in (**a**) precipitation (Prec), (**b**) daily minimum temperature (Tmin), and (**c**) daily minimum temperature (Tmax) by 2050 s relative to 1970–1999. 97 bias-corrected climate projections under four RCPs are used to characterize the uncertainty from climate models and emission scenarios. Only states where the constructed empirical models are significant at the 95% confidence level are included for analysis. Figure was created using software MATLAB 2015a (http://www.mathworks.com/).

**Figure 4:**
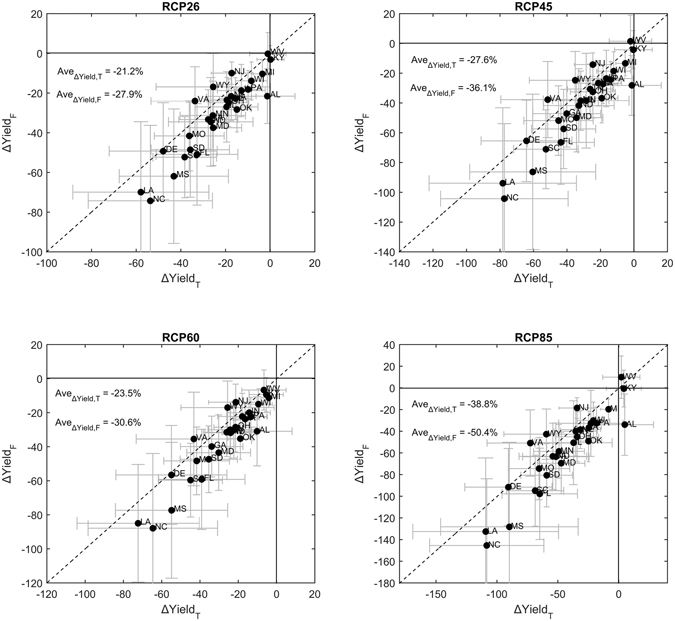
Projected state-level corn yield changes (%) by 2050 s relative to 1970–1999 under four RCPs estimated by the two crop models. The lower case T and F in the x and y labels indicate the estimates by crop model with and without considering the changes in crop growing pattern, respectively. The projected corn yields are estimated based on each climate model and the ensemble median value is shown with error bar denoting one standard deviation among the climate models in each RCP. The number indicate the multi-model ensemble median changes in corn yields averaged over investigated states with weights based on the long-term mean growing area. Figure was created using software MATLAB 2015a (http://www.mathworks.com/).

Previous empirical studies are based on the assumption that crop spatial distribution pattern is constant. How would this assumption add up to the uncertainty in projecting future climate impacts on crop yields? Here, climate change uncertainties are considered by utilizing 97 climate projections under four emission scenarios (Table S1). With global warming, growing season temperature is projected to increase for all investigated states while precipitation changes are more uncertain across the models and emission scenarios (Fig. 3). Figure 4 shows the projected corn yield changes by 2050 s (i.e. 2021–2050) based on the two crop models. Decrease of corn yields are found for almost all investigated corn growing states due to overwhelming effects of projected increase of Tmin and Tmax, suggesting that the majority of impacts will be driven by trends in temperature rather than precipitation. The smallest decrease is projected under RCP2.6 scenario, demonstrating the benefit of mitigations on reducing the negative climate impacts on corn yields. At the state level, the largest decrease is found in LA, which would lost all its crop yield under RCP8.5 scenario without adaptions. For those highly productive states, corn yield reduction by 20%~50% is projected depending on emission scenarios, which is in line with previous estimates^2, 38, 40^. To physically explain the difference in the estimates between the two crop models for all investigated states is not easy as it depends on the tradeoff among the considered climatic factors, the nonlinearity and etc. Importantly, based on the model trained with corn spatial distribution pattern changes, corn yield is projected to reduce by 10%~40%, which is about 10% less than that assuming fixed pattern. The results imply that previous empirical studies ignoring the changes of crop spatial distribution pattern would have overestimated the adverse climate impacts on corn yields. Further, the difference of estimated climate impacts by considering corn spatial distribution pattern changes is comparable to climate model spread, thereby introducing another important uncertainty source.

## Discussions

There are serval uncertainty sources in statistical crop models. For example, uncertainty is inherent in that empirical models are based on finite historical observations (referred to as sampling uncertainty). Here, this sampling uncertainty is estimated using bootstrap resampling technique. Specifically, 1000 bootstrap replicates of 30-year data points are generated for each crop–region combination and the statistical model is fitted for each replicate, based on which the sampling uncertainty is quantified by the standard deviation. Comparing Figs 2 and 4 shows that climate model uncertainty as indicated by the error bar is much larger than sampling uncertainty. Other aspects of crop model uncertainty mainly involve the variables and temporal scales selected for analysis. Indeed, to develop statistical models for describing climate-crop yield relations, we must struggle with the fundamental problem of variable selection. In this study, the models are developed from monthly averages of selected climate variables, and therefore does not explicitly consider other influencing factors such as extreme weathers^4, 5, 7, 41^. Further, climate anomalies occurring at different stages of crop development have varied impacts on agricultural production. However, including all possible months and variables will result in an over-parameterized model that tends to over-fit the training sample and give poor predictive performance^28^. Besides climate change, crop yields would be influenced by agricultural managements such as conservation tillage^42^, multiple cropping^43^, soil Mulching^44^, irrigation^45^ and fertilization^46, 47^, which are ignored in this study.

Certainly, the inability to establish significant relationships in some crop–state combinations imply that the methodology is not suitable in all locations. Therefore, more regionalized studies might improve the specifications of climate-crop yield relationships and may corroborate or refute the findings of this study. In this study, our models provide fairly accurate descriptions of historical corn yield variations, with more than 60% of corn yield anomalies explained for the country as a whole. The remaining variance of corn yields which cannot explained by the models suggest that the poorly understood processes such as crop infection, pollination, and dormancy may be important mechanisms through which climate conditions influences crop yields. We also emphasize that future projections are conditional on the assumption of no adaptations, and therefore are unlikely to represent the true future course of yield impacts. What’s more, we acknowledge that selection of reference year and gridded crop map are the uncertainty sources which would impact our results.

Therefore, we acknowledge that estimates of climate impacts in this study should not be interpreted as representing the correct magnitude of net climate change impacts. Rather, the results are intended to provide a measure of the sensitivity of climate impacts on crop yields to changes in crop spatial distribution pattern, which has received little attentions. Understanding the potential impacts in the absence of crop spatial distribution pattern is a critical step towards planning and prioritizing effective adaptation options. Moreover, our analysis is restricted to the state level as we only consider the transient crop spatial distribution pattern changes between counties within a specific state. That is, corn growing location within a specific county remain constant inheriting from the fixed gridded corn area map. Thus, we emphasize the need to develop finer scale crop area datasets for constraining the estimations in the future.

## Conclusions

The impacts of climate change on crop yield have been extensively studied over the past few decades, but with limited attention on the effects of changes in crop spatial distribution pattern. In this study, we explore how changes in county-level corn spatial distribution pattern modulate the response of its yields to climate change at the state level over the CONUS.

We found that changes in crop spatial distribution pattern affect the assessment of climate change impacts on corn yields. Changes in county-scale crop spatial distribution pattern can modulate the state-level corn yield response to climate change, with distinct impacts on the magnitude and even the direction at the state level. Averaged over the country, climate change has led to 2.5% decrease of corn yield for the historical period, which is reduced to 1.8% if accounting for the effects of corn growing pattern changes. The benefits are mainly achieved by reducing the adverse impacts of daily maximum temperature and strengthening the positive effects of precipitation. What’s more, changes in crop spatial distribution pattern have played a more profound role in modulating the linear climatic effects than the non-linear climatic effects. Under future climate change scenarios, corn yields are projected to decrease 20~50% depending on emission scenarios, which could be reduced by ~10% after accounting for crop growing pattern changes.

Overall, our results indicate that previous empirical studies ignoring the changes in crop growing pattern would have overestimated the adverse climate impacts on corn yield over CONUS. The difference between estimated climate impacts with and without considering corn spatial distribution pattern is even comparable to the range among climate models, thereby introducing another important uncertainty source. A focus assuming constant spatial distribution pattern may therefore bias assessments of the vulnerability of agriculture to climate change. As a result, the effects of crop spatial distribution pattern are important to consider in impact studies, as they may affect both estimates of impacts as well as associated estimates of uncertainty. Despite the consensus that warming will likely have adverse impacts on agriculture in tropic and sub-tropic regions, there is continuing debates on whether warming will be a net loss or gain for agriculture in temperate regions like CONUS^48–50^. By revealing the important least-understood uncertainty source from crop spatial distribution pattern changes, this study complement previous efforts for better understanding the link between weather and yields for crops grown in CONUS.

## Materials and Methods

### Crop census data and climate conditions

County-level corn yield and harvest area are obtained from the US Department of Agriculture (USDA) National Agriculture Statistics Survey’s Quick Stats database (http://www.nass.usda.gov/Quick_Stats). Historical climate data during 1970–1999 is based on the gridded observations, which include precipitation (Prec), daily minimum (Tmin) and maxim air temperature (Tmax) at 0.125 grid resolutions across the CONUS^51^. We also use future climate projections under four Representative Concentration Pathway (RCP) scenarios (RCP2.6, RCP4.5, RCP6.0 and RCP8.5) for 2021–2050. The climate projections are statistically downscaled to 1/8 degree resolution and bias-corrected against the observed climate over CONUS using the bias-correction and spatial-downscaling approach (BCSD)^52, 53^ and are obtained from ftp://gdo-dcp.ucllnl.org/pub/dcp/archive/cmip5/hydro. There are a total of 97 climate projections under four RCPs. We didn’t rely on certain criteria to select a subset of climate projections. Instead, we used all the 97 climate projections in this study (Supplementary Table S1), which cover the largest possible range of CMIP5 climate projections.

### Methodology

To get the background information on the changes in corn spatial distribution pattern, we develop a stability index (*SI*_*s*_) by:

1 $$S{I}_{s}=\frac{{\sum }_{c}^{m}{({A}_{c,y}^{usda}-{A}_{c,y0}^{usda})}^{2}}{{\sum }_{c}^{m}{A}_{c,y0}^{usda}/m}\,\,c=1\ldots m$$

Where ${A}_{c,y}^{usda}\,$is the total corn area for county *c* from USDA census. *s* is the state, *y* is for the year while *y*0 is the reference year 2000. Larger *SI*_*s*_ means larger instability (changes) of crop spatial distribution pattern between counties within this state and vice versa. It should be noted that crop yield stability is a broad topic and there are generally two dimensions involved when referring to yield stability: its temporal and spatial stability. As for the temporal stability, the standard deviation and the coefficient of variation (CV) are commonly used index to measure yield stability over time^20, 36, 54^. There are also more complex index for measuring the temporal stability of crop yield, e.g. the ecovalence index, the stability variance index and the yield stability index^55^. Here, we refers to the stability of crop spatial distribution pattern and the evolutions of spatial stability as indicated by the index is shown in Fig. 1b.

To investigate the effects of crop spatial distribution change, we follows the logic of statistical modeling of crop yield response to climate conditions as follows. Typically, one of the key step in constructing the statistical model is to aggregate the gridded climate data into the administrative level, at which crop yield census is reported^26, 28, 35^. This aggregation is conducted based on the weights given by a gridded crop area map, assuming that the relative distribution of crops in space has remained constant over the study period. We noticed that there exist time series of crop area data at the county scale in CONUS, which enable us to investigate the effects of crop spatial distribution pattern changes at the state level. In this study, two statistical models are fitted for each state, with one based on the newly generated transient crop maps and the other based on a fixed crop map. We note that there are potentially other approaches to explore the effects of crop spatial distribution pattern changes, e.g. incorporating the stability index into the statistical model. If choosing this approach, however, we may have to struggle with the definition of stability index and evaluate which stability index can better represent crop spatial distribution pattern changes and the associated uncertainties. This is evidently not within the scope of this study as this index is developed with the purpose of showing the evolution of crop spatial distribution pattern for each growing state, which is used to justify the following investigation of crop pattern change effects.

Here, we use the gridded corn growing map from MIRCA2000^30^ as the basis to develop the transient crop growing maps as follows: first, the gridded corn area are scaled by the ratio between the USDA reported county corn areas and the sum of gridded corn area within each county such that the sum of gridded corn area match the USDA census at the county scale.

2 $${A}_{c,i,y0}^{\text{'}}={A}_{c,i,y0}\ast \frac{{A}_{c,y0}^{usda}}{{\sum }_{i}^{n}{A}_{c,i,y0}}\,\,i=1\ldots n$$

Where ${A}_{c,i,y0}^{\text{'}}$ and *A*_*c,i,y*0_ the rescaled and original crop area for grid *i* in county *c* for year *y*, ${A}_{c,y0}^{usda}\,$ is the total crop area for county *c* from USDA census. We choose the year 2000 as the reference year (*y*0) since the gridded map represent the conditions around 2000. We acknowledge that the results may depend on the selection of the reference year and gridded crop map. About 6% of mismatches are found between USDA census and gridded map and excluded in our analysis. Second, the time series of corn growing maps (${A}_{c,i,y}^{\text{'}}$) was developed by multiplying the ratios of USDA crop area to the reference USDA crop area.

3 $${A}_{c,i,y}^{\text{'}}={A}_{c,i,y0}^{\text{'}}\frac{{A}_{c,y}^{usda}}{{A}_{c,y0}^{usda}}\,\,y=1970\ldots 1999$$

By this, information on the changes in corn growing pattern between counties is incorporated. It should be noted that corn growing location within a specific county remain constant inheriting from the gridded corn area map since no dynamic grid scale maps are available. Thus, we investigate the effects of crop pattern changes between counties in modulating climate impacts on corn yield at the state level.

Three climatic variables, i.e., the growing season (June, July and August) mean precipitation (Prep), daily minimum temperature (Tmin) and daily maximum temperature (Tmax) are used for investigating the first-order climate impacts on crop yields. The gridded climate data is aggregated to the state level for 1970–1999 based on the two sets of maps, i.e. one using the transient weights incorporating crop spatial distribution pattern changes and the other based on the fixed crop map at the year 2000. The state-level climate based on transient crop maps differ not only in the mean but also in the temporal variability from that on a fixed crop map (Supplementary Table S2). Then multiple regressions are constructed using time series of state-level climate as predictor and corn yields as predictand as follows:

4 $$\begin{array}{rcl}Yiel{d}_{s,y} & = & {\beta }_{0}+{\beta }_{1}Pre{c}_{s,y}+{\beta }_{2}Pre{c}_{s,y}^{2}\,+{\beta }_{3}Tmi{n}_{s,y}+{\beta }_{4}Tmi{n}_{s,y}^{2}\\  &  & +\,{\beta }_{5}Tma{x}_{s,y}+{\beta }_{6}Tma{x}_{s,y}^{2}\,+{\alpha }_{s}+{\varepsilon }_{s,y}\end{array}$$

Where *β*_0–5_ are the model parameters, *α*_*s*_ is the random intercept term representing the constant regional effects, *ε*_*s,y*_ is for the error term, *s* is for the state and *y* is for the year. The coefficients of statistical models for each investigated state are provided in Supplementary Table S3.

Four distinct features of the regression models are considered: (1) To remove out non-weather effects such as technological improvements, the linear trend of time series were removed based the least squares method (note: the obtained results are similar using the other widely used approach, i.e. deriving the difference from one year to the next). That is, the empirical relations are constructed between the anomalies of yield and climate; (2) another important step when analyzing sources of yield variation is to account for any autocorrelation present in crop yields. As shown in the Supplementary Figure S1, in 89.6% of investigated counties (gray colors), the autocorrelation assumption of corn yields does not hold at the at the 5% significance level. In those areas where autocorrelation exist (10.4% of investigated counties, yellow colors), an autoregressive model was fit and the number of years of previous yields to include was determined through Akaike Information Criteria (AIC) to find the model with the best balance between yield prediction and simplicity following the approach by Lobell *et al*.^56^; (3) Since the linear relations may not hold under all conditions due to the nonlinear response of crop growth to climate, we consider the quandary terms for each selected climate variable to account for the nonlinear effects; (4) To estimate the uncertainty associated with the crop models, a bootstrap resampling technique was used. Specifically, the 30-year time series of datasets (i.e. 30 points) are resampled to create 1000 different datasets of 30 points, and the statistical model is fitted for each data set. Bootstrap sampling is well adopted in statistical models linking climate and crop yields^1, 20, 35^. The MATLAB code for bootstrap sampling technique can be found at: https://www.mathworks.com/help/stats/bootstrp.html. The standard deviation of the results based on these samples are used to indicate the crop model uncertainty ranges.

Here, we validate the statistical crop model based on the statistical significance (i.e. *P* value) and the model’s explanatory power as measured by the coefficient of determination (R^2^). A low coefficient of determination (R^2^) indicates the poor model performance in capturing the observed crop yield response to climate^28^. Only those models that are statistically significant at the 95% confidence level (i.e. *P* < 0.05) are included for analysis. And the coefficient of determination (R^2^) is provided in Table 1. We note that there are other ways to validate the statistical model, e.g. training the model with a subset of years and validating it using the remaining years of data^2^. The model performance are then quantified by means of average error and/or correlation between predicted and ‘actual’ yields and etc. However, issues would occur as to which subset of 30-year census data should be used (e.g. 15 years) and the length of subset data would not necessarily meet the requirement of adequate power in the statistical regressions. In this study, we use the entire time series of crop yield and climate data to ensure adequate power of statistical relations and the approach we choose for model validation is well adopted and documented in statistical modeling of climate-crop yield relations^4, 28, 32, 35, 36^. Here, the statistical models are fitted for a total of 41 states. The statistical significance of the regressions is calculated according to the two-tailed Student’s t-test. In 13 states, either one or both models are not statistically significant. Only those states (a total of 28 states, see Table 1) where both models are statistically significant are selected for analysis. By comparing the results based on the two regression models, the effects of crop spatial distribution pattern changes between counties on state-level corn yield response to climate change can be quantified.

We predict future changes in corn yields under a range of precipitation and temperature anomaly scenarios by 2050 s. We limit our projections to 2050 s because temperatures beyond this period are frequently beyond the range of historical temperatures used to fit the statistical models. Changes in Prec, Tmin and Tmax are computed for each of the 97 model simulations with weights based on the MIRCA2000 gridded corn area map and applied to the statistical model. Future crop yield estimates are compared to historical estimates, with inter-model standard deviation to indicate the range among climate models. Comparing the projected changes using the two statistical models has great implications for understanding the uncertainties in assessing future climate change impacts on crop yields.

## Electronic supplementary material

Supplementary materials
