# Supplementary material for: Crop yield response to climate change varies with crop spatial distribution pattern
Source: Sci Rep. 2017 May 3;7:1463. doi: 10.1038/s41598-017-01599-2 (PMC5431105; doi:10.1038/s41598-017-01599-2)
Supplement: Supplementary file 1 — Supplementary materials [file 41598_2017_1599_MOESM1_ESM.pdf]

Crop yield response to climate change varies with crop spatial distribution pattern

Guoyong Leng[1,*] and Maoyi Huang[2]

[1]Joint Global Change Research Institute, Pacific Northwest National Laboratory, College Park MD 20740, USA

[2]Earth System Analysis and Modeling Group, Atmospheric Sciences & Global Change Division, Pacific Northwest National Laboratory, USA

*Corresponding author address: Guoyong Leng, Joint Global Change Research Institute, Pacific Northwest National Laboratory, College Park MD, 20740.
E-mail: Guoyong.Leng@pnnl.gov

Supplementary Materials

Table S1 Climate models and emission scenarios used in this study.

Table S2 Difference (%) of state-level climate in the mean and inter-annual variability based on transient crop maps relative to that based on fixed crop maps.

Table S3 The coefficients of statistical models for each investigated state. The numbers underlined indicate the coefficients of statistical model considering corn area distribution changes, while the regular numbers denote the model assuming fixed corn area distribution.

Figure S1 County-level distribution of autocorrelation of corn yield from the Durbin-Watson test. Gray colors show where the autocorrelation assumptions are violated at $p>0.05$ and yellow colors where they hold at $p\leq0.05$. Figure was created by NCAR Command Language[1]

Table S1 Climate models and emission scenarios used in this study

ID	Climate Model	Emission Scenarios			
1	access1-0		rcp45		rcp85
2	bcc-csm1-1	rcp26	rcp45	rcp60	rcp85
3	bcc-csm1-1-m		rcp45		rcp85
4	canesm2	rcp26	rcp45		rcp85
5	ccsm4	rcp26	rcp45	rcp60	rcp85
6	cesm1-bgc		rcp45		rcp85
7	cesm1-cam5	rcp26	rcp45	rcp60	rcp85
8	cmcc-cm		rcp45		rcp85
9	cnrm-cm5		rcp45		rcp85
10	csiro-mk3-6-0	rcp26	rcp45	rcp60	rcp85
11	fgoals-g2	rcp26	rcp45		rcp85
12	fio-esm	rcp26	rcp45	rcp60	rcp85
13	gfdl-cm3	rcp26	rcp45	rcp60	rcp85
14	gfdl-esm2g	rcp26	rcp45	rcp60	rcp85
15	gfdl-esm2m	rcp26	rcp45	rcp60	rcp85
16	giss-e2-h-cc		rcp45		
17	giss-e2-r	rcp26	rcp45	rcp60	rcp85
18	giss-e2-r-cc		rcp45		
19	hadgem2-ao	rcp26	rcp45	rcp60	rcp85
20	hadgem2-cc		rcp45		rcp85
21	hadgem2-es	rcp26	rcp45	rcp60	rcp85
22	inmcm4		rcp45		rcp85
23	ipsl-cm5a-mr	rcp26	rcp45	rcp60	rcp85

24	ipsl-cm5b-lr		rcp45		rcp85
25	miroc-esm	rcp26	rcp45	rcp60	rcp85
26	miroc-esm-chem	rcp26	rcp45	rcp60	rcp85
27	miroc5	rcp26	rcp45	rcp60	rcp85
28	mpi-esm-lr	rcp26	rcp45		rcp85
29	mpi-esm-mr	rcp26	rcp45		rcp85
30	mri-cgcm3	rcp26	rcp45		rcp85
31	noresm1-m	rcp26	rcp45	rcp60	rcp85
Number of Projections	97	31	31	16	29

Table S2 Difference (%) of state-level climate in the mean and inter-annual variability based on transient crop maps relative to that based on fixed crop maps.

State name	Mean	Variability
AL (Alabama)	-1.37	0.78
DE (Delaware)	-0.03	-0.74
FL (Florida)	-0.81	-5.73
GA (Georgia)	5.41	4.26
IL (Illinois)	-0.50	1.42
IN (Indiana)	0.09	-0.06
IA (Iowa)	0.23	-0.35
KY (Kentucky)	2.00	2.67
LA (Louisiana)	0.15	-2.85
MD (Maryland)	0.35	0.15
MI (Michigan)	0.17	-0.33
MN (Minnesota)	-0.48	3.19
MS (Mississippi)	-10.37	6.36
MO (Missouri)	0.71	-3.39
NE (Nebraska)	0.88	-4.70
NJ (New Jersey)	0.03	-1.47
NC (North Carolina)	4.91	-10.34
ND (North Dakota)	-2.80	9.35
OH (Ohio)	0.57	1.07
OK (Oklahoma)	-12.70	1.44
PA (Pennsylvania)	-0.52	-0.98
SC (South Carolina)	1.33	2.14
SD (South Dakota)	-3.28	3.69
TN (Tennessee)	1.89	1.17
VA (Virginia)	1.98	6.90
WV (West Virginia)	-0.29	8.79
WI (Wisconsin)	-0.63	2.76
WY (Wyoming)	0.32	-3.03

Table S3 The coefficients of statistical models for each investigated state. The numbers underlined indicate the coefficients of statistical model considering corn area distribution changes, while the regular numbers denote the model assuming fixed corn area distribution.

State name	β_1	β_2	β_3	β_4	β_5	β_6
AL (Alabama)	7.90	-0.24	8.44	10.86	-15.42	-5.11
	5.89	-0.60	3.49	5.64	-15.65	-4.45
DE (Delaware)	5.19	-2.90	8.96	-2.66	-20.28	-5.31
	2.72	-3.18	9.42	-3.00	-22.20	-5.82
FL (Florida)	-2.10	3.29	3.30	-0.13	-9.06	-11.72
	-6.58	4.01	1.91	-5.26	-12.97	-12.47
GA (Georgia)	7.84	0.87	-3.63	0.62	-9.29	-4.24
	6.76	0.54	-4.81	1.32	-10.34	-4.50
IL (Illinois)	11.28	-0.53	-15.92	5.69	9.39	-5.78
	9.96	-0.64	-16.39	5.64	8.06	-6.32
IN (Indiana)	9.60	-0.82	-9.70	6.84	3.73	-5.00
	7.61	-0.72	-9.35	6.59	1.76	-5.57
IA (Iowa)	3.03	-4.47	2.71	-0.15	-7.78	-0.34
	1.33	-4.51	1.93	-0.18	-9.27	-0.99
KY (Kentucky)	9.29	-5.66	-1.33	6.17	-4.76	-2.53
	7.02	-5.72	-0.28	7.08	-7.02	-2.87
LA (Louisiana)	6.08	0.03	-16.21	-4.95	5.10	-8.73
	6.39	0.79	-26.00	-8.67	10.84	-8.13
MD (Maryland)	4.41	0.04	9.96	-0.73	-18.19	-1.78
	1.26	0.05	13.09	-2.36	-22.49	-2.52
MI (Michigan)	10.77	-9.01	3.21	0.03	-3.03	-0.69
	8.49	-8.90	3.53	0.19	-5.53	-1.29
MN (Minnesota)	2.89	-13.10	1.14	-4.59	-5.00	0.38
	1.12	-13.49	0.53	-4.46	-6.02	-0.25

MS (Mississippi)	8.03	-3.89	-8.57	-4.99	2.74	-4.66
	7.14	-3.04	-13.82	-8.35	4.99	-6.37
MO (Missouri)	3.75	-4.33	4.36	-7.27	-15.28	2.80
	3.33	-4.43	2.36	-6.65	-15.08	1.97
NE (Nebraska)	4.84	-7.28	-1.17	-4.94	-1.91	1.79
	3.56	-7.00	-0.76	-4.72	-3.62	1.18
NJ (New Jersey)	13.15	-5.26	-1.77	2.97	-4.53	-5.47
	11.64	-8.11	3.14	4.43	-7.40	-4.56
NC (North Carolina)	4.97	3.29	0.33	-2.13	-16.95	-7.66
	1.14	1.83	-0.44	-3.24	-20.65	-10.63
ND (North Dakota)	5.62	-11.31	1.95	-2.78	-1.62	-2.51
	2.30	-16.96	3.48	-3.47	-5.85	-2.48
OH (Ohio)	8.78	0.90	-6.83	2.17	-0.99	-2.50
	7.31	1.06	-7.60	2.22	-2.43	-3.12
OK (Oklahoma)	2.40	9.23	-1.69	1.10	-3.01	-2.13
	2.79	12.99	-7.74	0.22	-1.59	-2.82
PA (Pennsylvania)	14.37	-9.10	3.22	1.85	-7.89	-2.27
	13.66	-11.26	3.35	1.98	-9.17	-2.56
SC (South Carolina)	8.92	-0.72	-7.74	-1.12	-15.45	-1.85
	8.04	-1.01	-10.97	-3.66	-15.85	-2.23
SD (South Dakota)	-8.93	-20.79	3.86	-5.37	-12.93	1.30
	-10.39	-20.85	2.20	-5.27	-14.34	0.27
TN (Tennessee)	10.45	-8.07	-5.05	0.00	-3.08	-0.78
	4.13	-8.30	-1.69	1.64	-8.53	-1.92
VA (Virginia)	14.64	3.24	-1.30	1.86	-9.84	-6.74
	8.94	4.23	5.81	6.68	-15.91	-8.32

WV (West Virginia)	10.05	1.85	-7.55	10.53	-1.85	-5.47
	1.14	2.81	-4.05	13.62	-7.35	-5.83
WI (Wisconsin)	8.61	-8.50	-4.14	0.58	5.47	-2.63
	6.87	-8.51	-3.93	0.62	3.69	-3.23
WY (Wyoming)	16.35	0.26	4.47	-14.90	6.17	1.79
	12.29	2.26	4.42	-8.44	3.85	-0.29

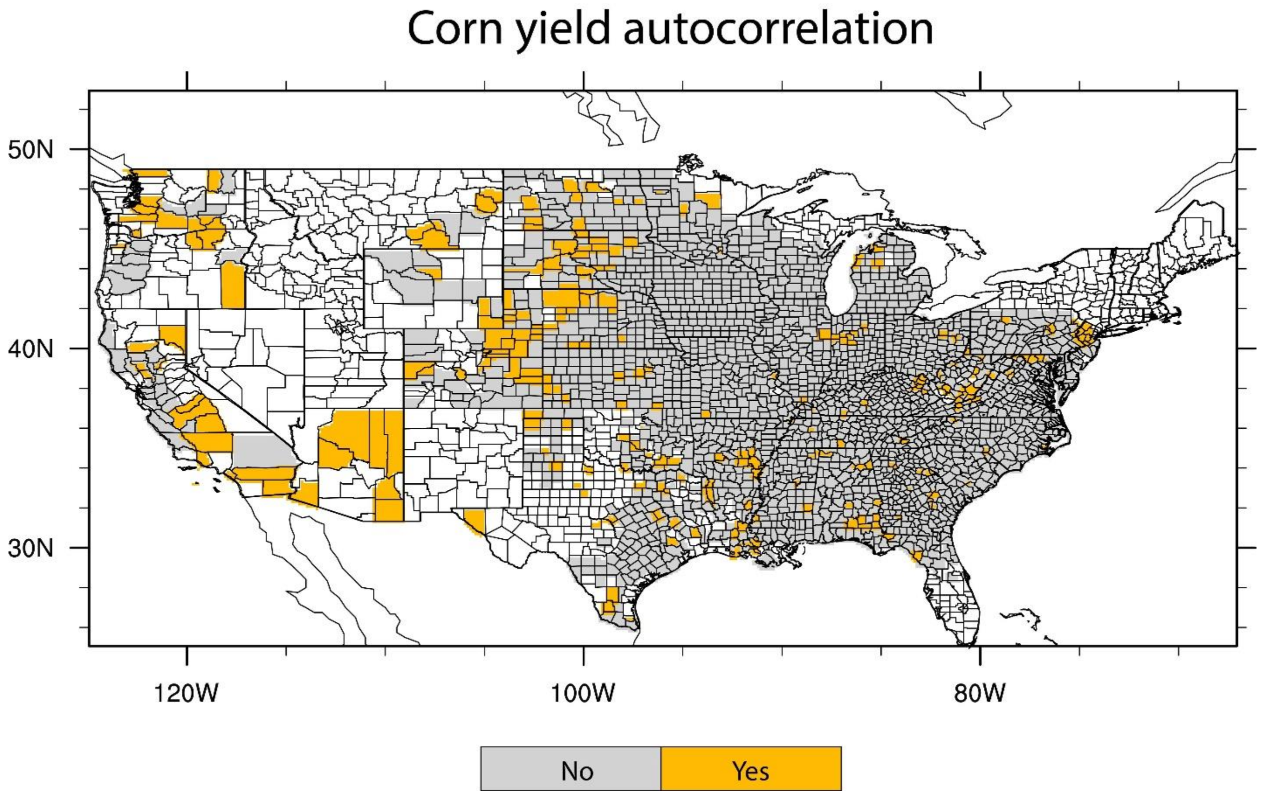

Figure S1 County-level distribution of autocorrelation of corn yield from the Durbin-Watson test. Gray colors show where the autocorrelation assumptions are violated at $p>0.05$ and yellow colors where they hold at $p \leq 0.05$. Figure was created by NCAR Command Language[1]

References

1. The NCAR Command Language (Version 6.1.2) [Software]. (2013). Boulder, Colorado: UCAR/NCAR/CISL/VETS. http://dx.doi.org/10.5065/D6WD3XH5.
